# Ability of a Machine Learning Algorithm to Predict the Need for Perioperative Red Blood Cells Transfusion in Pelvic Fracture Patients: A Multicenter Cohort Study in China

**DOI:** 10.3389/fmed.2021.694733

**Published:** 2021-08-16

**Authors:** Xueyuan Huang, Yongjun Wang, Bingyu Chen, Yuanshuai Huang, Xinhua Wang, Linfeng Chen, Rong Gui, Xianjun Ma

**Affiliations:** ^1^Department of Blood Transfusion, The Third Xiangya Hospital, Central South University, Changsha, China; ^2^Department of Blood Transfusion, The Second Xiangya Hospital, Central South University, Changsha, China; ^3^Department of Transfusion, Zhejiang Provincial People's Hospital, Hangzhou, China; ^4^Department of Transfusion, The Affiliated Hospital of Southwest Medical University, Luzhou, China; ^5^Department of Transfusion, Beijing Aerospace Center Hospital, Beijing, China; ^6^Department of Transfusion, Beijing Shijitan Hospital, Capital Medical University, Beijing, China; ^7^Department of Blood Transfusion, Qilu Hospital of Shandong University, Jinan, China

**Keywords:** pelvic fracture, perioperative, RBCs transfusion, predictive model, machine learning

## Abstract

**Background:** Predicting the perioperative requirement for red blood cells (RBCs) transfusion in patients with the pelvic fracture may be challenging. In this study, we constructed a perioperative RBCs transfusion predictive model (ternary classifications) based on a machine learning algorithm.

**Materials and Methods:** This study included perioperative adult patients with pelvic trauma hospitalized across six Chinese centers between September 2012 and June 2019. An extreme gradient boosting (XGBoost) algorithm was used to predict the need for perioperative RBCs transfusion, with data being split into training test (80%), which was subjected to 5-fold cross-validation, and test set (20%). The ability of the predictive transfusion model was compared with blood preparation based on surgeons' experience and other predictive models, including random forest, gradient boosting decision tree, K-nearest neighbor, logistic regression, and Gaussian naïve Bayes classifier models. Data of 33 patients from one of the hospitals were prospectively collected for model validation.

**Results:** Among 510 patients, 192 (37.65%) have not received any perioperative RBCs transfusion, 127 (24.90%) received less-transfusion (RBCs < 4U), and 191 (37.45%) received more-transfusion (RBCs ≥ 4U). Machine learning-based transfusion predictive model produced the best performance with the accuracy of 83.34%, and Kappa coefficient of 0.7967 compared with other methods (blood preparation based on surgeons' experience with the accuracy of 65.94%, and Kappa coefficient of 0.5704; the random forest method with an accuracy of 82.35%, and Kappa coefficient of 0.7858; the gradient boosting decision tree with an accuracy of 79.41%, and Kappa coefficient of 0.7742; the K-nearest neighbor with an accuracy of 53.92%, and Kappa coefficient of 0.3341). In the prospective dataset, it also had a food performance with accuracy 81.82%.

**Conclusion:** This multicenter retrospective cohort study described the construction of an accurate model that could predict perioperative RBCs transfusion in patients with pelvic fractures.

## Introduction

Pelvic fracture is a condition caused by high-energy trauma that is often accompanied by multiple injuries. It accounts for ~3% of all fracture injuries (1). Patients with pelvic fractures have an overall high injury severity score, which indicates the serious injury (2–4). Due to rapid bleeding and difficulty in stopping the bleeding, the mortality rates are high, reaching up to 30% in hemodynamically unstable pelvic fracture patients. In addition, the severity of the injury, the complexity of the fracture, and the surrounding neurovascular anatomical structure result in very high perioperative blood loss and allogenenic blood transfusion (ABT) rates in patients with pelvic fractures (5, 6).

Allogeneic red blood cells (RBCs) transfusion may increase the risk of complications during surgery and cause serious adverse reactions (7). A recent study reported that 166 patients who received ABT had serious complications, and 26 of them died (8). ABT is an independent risk factor for perioperative morbidity and mortality (9, 10). However, during the initial stages of trauma and preoperative blood preparation, it is difficult to predict the perioperative requirement for RBCs transfusion in patients with pelvic fracture. RBCs transfusion is currently primarily based on the surgeons' experience and on hemoglobin (Hb) concentration (11). As RBCs transfusion solely based on Hb levels is regarded as one-sided and incorrect, accurate method is needed to assist perioperative blood management (PBM) in patients with pelvic fracture. This method should reduce the wasting of blood resources, reduce the morbidity of transfusion-related adverse reactions, and improve patient prognosis. To the best of our knowledge, no reports to date have described a method that can accurately predict the risk and scope of RBCs transfusion during surgery of pelvic fracture.

Machine learning, an application in artificial intelligence, is a scientific discipline that studies the regularities of related data through computer learning. Machine learning has been widely used in multiple fields, such as computer vision, language recognition, and robot control (12). Research and practice in biomedicine have also benefited from machine learning (13–17). For example, an extreme gradient boosting (XGBoost) algorithm, a scalable machine learning system for tree boosting, has particular advantages in machine learning methods. This algorithm has shown an ability to process missing values, utilize data scaling, thus, successfully processing computationally valid variants (18–20).

In this study, an XGBoost-based machine learning model was constructed using clinical and laboratory data from multiple Chinese centers to accurately predict the need (no-transfusion, less-transfusion or more-transfusion) for perioperative RBCs transfusion in patients with pelvic fracture.

## Materials and Methods

### Trial Design and Participants

**Figure 1:**
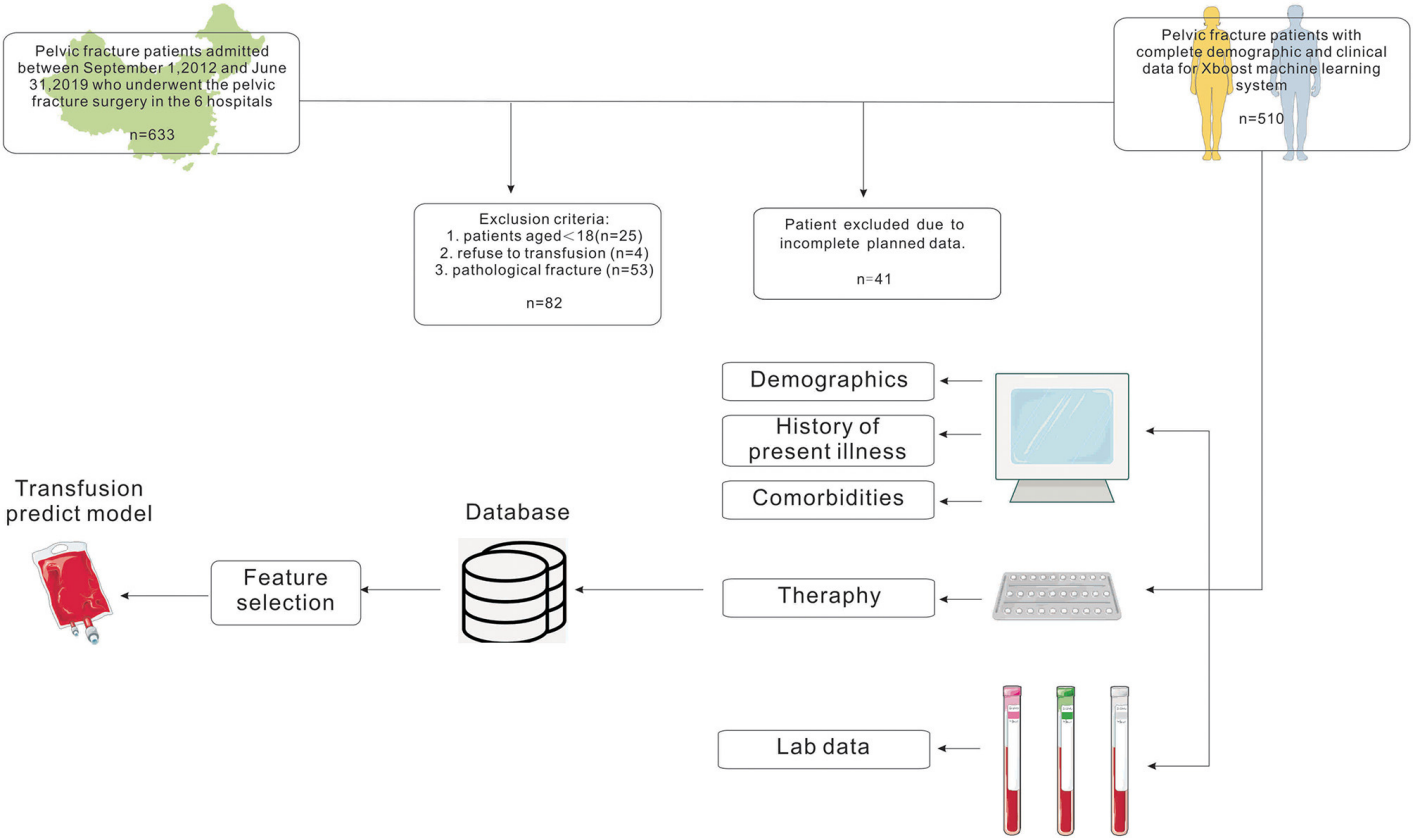
Flow chart showing the inclusion and exclusion criteria of patients and model-making process for the study.

This study was conducted at the six following centers in China between September 2012 and June 2019: the Third Xiangya Hospital of Central South University, the Second Xiangya Hospital of Central South University, Zhejiang Provincial People's Hospital, Affiliated Hospital of Southwest Medical University, Beijing SHIJITAN Hospital, and Aerospace Center Hospital. The subjects were patients who underwent surgery for pelvic fractures in these centers. Patients aged <18 years, patients who refused transfusion, and patients with pathologic pelvic fracture were excluded. We finally included 510 cases with complete data (Figure 1). The perioperative period was defined as 7 days before surgery to 7 days after surgery.

The study protocol was approved by the Institutional Review Board of the Third Xiangya Hospital of Central South University (NO: 2019-S009) and was registered at www.ClinicalTrials.gov (NCT03855644).

Data of pelvic fracture surgery patients who underwent surgery in the Third Xiangya Hospital of Central South University between May 1st 2021, and May 20th 2021 were prospectively collected to further validate the model.

### Data Collection

All the variables in this study were retrospectively collected from the electronic medical recording system of each center. A total of 107 variables were collected; variables that were missing for more than 20% of patients were not analyzed. Forty-four variables were included in the correlation analysis, and variables with correlation coefficients >0.5 were not further analyzed according to feature important score (FIS). The correlation coefficient refers to an association between variables. The Pearson correlation coefficient was typically used to compare normally distributed data. For continuous data with non-normal distribution, for ordinal data, or data with relevant outliers, a Spearman rank correlation was used to measure the association. FIS is the feature importance evaluation that comes with XGBoost. The FIC weighs the average importance of each feature at the model level. A total of 17 variables were analyzed, including demographic and clinical characteristics such as cause of fracture (traffic, grind, fall, and others), type of fracture (Tile type), site of fracture (pubic, sacrum, ankle joint, acetabular, iliac ring), Injury Severe Score (ISS score), the occurrence of hemorrhagic shock, volume replacement therapy (hydroxyethyl starch injection, HES injection), iron therapy and hemostasis. Laboratory variables included hematocrit (HCT, %) and preoperative Hb concentration (g/L), preoperative mean arterial pressure (MAP, mmHg), total serum protein (U/L), aspartate transaminase (AST, U/L), and partial pressure of carbon dioxide (PaCO_2_, mmHg). Surgical variables included time from injury to the first operation (TIFO, day), and intraoperative cell salvage (ml). Other factors included organ damage.

Hemostasis treatment was defined as perioperative treatment with tranexamic acid or white eyebrow venom hemagglutinin. Iron therapy was defined as perioperative intravenous injection of ferrous sulfate or iron sucrose or oral administration of ferrous succinate. Hemorrhagic shock was defined as blood pressure below 90/60 mmHg caused by blood loosed. Intraoperative cell salvage was defined as patients who received the blood transfusion from the same patient's blood loss by anticoagulation, salvage, filtration, and washing. MAP = (systolic blood pressure + 2 ^*^ diastolic blood pressure)/3; TIFO was defined as the time from the trauma that caused the fracture to the first operation.

### Data Set Processing

Patients were divided into three categories according to the different RBCs transfusion strategies. The no-transfusion group included patients who did not receive perioperative transfusions of allogeneic RBCs; the less-transfusion group included patients who were received with allogeneic RBCs < 4U; and the more-transfusion group included patients who were received with allogeneic RBCs ≥ 4U.

The patients were randomly divided into a training subset, which included 80% of patients, and a test subset, which included the remaining 20%, such that three classifications were maintained across both the training and test subsets. We used the XGBoost algorithm to find the relationship between variables and outcome. Five-fold cross-validation was performed taking into consideration the limited sample size (21), randomly splitting the dataset into 5 subsets, and using them in each iteration, four of them to train the models and the last one for validation. After five iterations, each subset was validated and the validation results were combined to robustly assess the model performance.

### Statistical Analysis

The machine learning based on XGBoost algorithms was compared with blood preparation based on surgeons experience and other predictive models, including random forest, gradient boosting decision tree, K-nearest neighbor, logistic regression, and Gaussian naïve Bayes classifier models using index accuracy, Youden index, Kappa coefficient, the area under the receiver operating characteristic curve (AUC) and the associated 95% confidence interval (CI). Feature ranking was obtained by computing Shapley Additive Explanation values (SHAP values) (22). Accuracy was calculated as the total number of categories predicted correctly divided by the total number of test set samples (Accuracy = the number of samples whose class was predicted correctly/the total number of samples). The Youden index was a type of index measure that combined sensitivity and specificity to evaluate the authenticity of a predictive model. The Youden index was defined as J(t) = sensitivity (t) + specificity (t) −1. The AUC was the area under the receiver operating characteristic (ROC) curve that assessed the accuracy of the model. The Kappa coefficient was a measure of the consistency between a predicted category and an actual category, based on linear weighting; the formula was as follows.

The kappa coefficient is a function of two quantities: the observed percent agreement.

$$P_{o}=\sum_{i=1}^{k}p_{ii}$$

$$\begin{array}{l} P_{e}=\sum_{i=1}^{k}p_{i}+p_{+i,} \end{array}$$

which is the value of the observed percent agreement under statistical independence of the classifications. The observed percent agreement is generally considered artificially high. It is often assumed that it overestimates the actual agreement since some agreement may simply occur due to chance. The kappa coefficient is given by,

$$\begin{array}{l} k=\frac{P_{o}-P_{e}}{1-P_{e}} \end{array}$$

Continuous variables were expressed as the mean with range or median with interquartile range (IQR), compared by ANOVA, while categorical variables as counts (percentages) and by the Pearson χ^2^ test. Data that could not be analyzed by these methods were evaluated by Kruskal–Wallis analysis. A *p*-value <0.05 was considered statistically significant. Interaction analysis was performed to assess the effects of different variables on changes in transfusion risk.

## Results

### Numbers Analyzed

**Figure 2:**
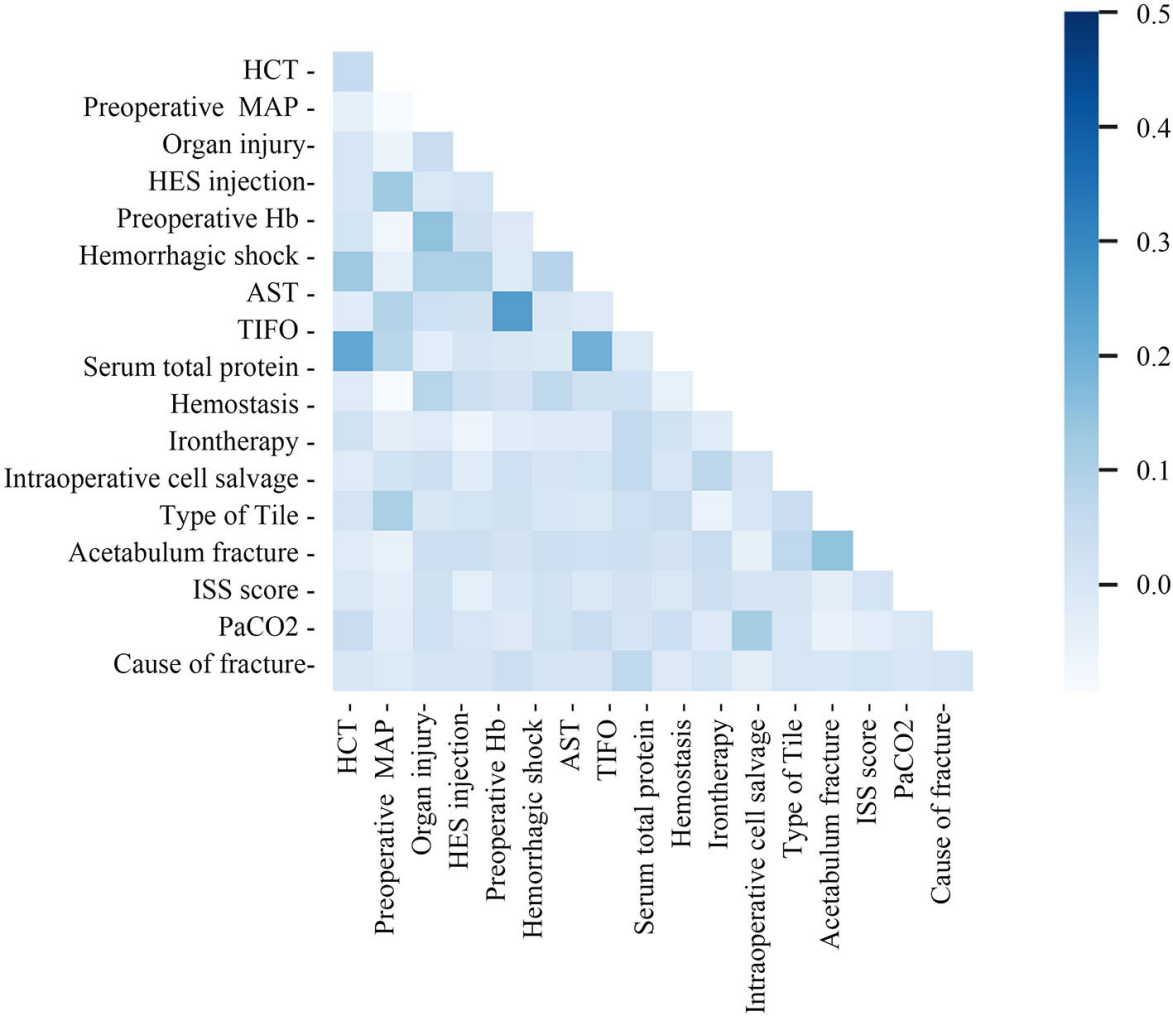
Correlation matrix of features included within machine learning algorithms in transfusion predictive model. MAP, preoperative mean arterial pressure; TIFO, time from the injury and the first operation; AST, aspartate transaminase; HCT, hematocrit; ISS, injury severe score; PaCO_2_, partial pressure of carbon dioxide; HES, hydroxyethyl starch.

The study cohort consisted of 510 patients, 408 allocated to the training set and 102 to the test set (Figure 1). Seventeen variables were included in the optimization model, with correlation analyses between variables performed to determine the independence of each variable (Figure 2). Table 1 shows the 17 model variables in the patients with pelvic fractures. Of the 510 patients, 192 (37.6%) have not received any RBCs transfusions, 127 (24.9%) received <4U of RBCs, and 191 (37.5%) received ≥4U of RBCs transfusion during the perioperative period, which was classified in no-transfusion group, less transfusion group, and more transfusion group, respectively. Using traditional statistical analyses, we found that some of the variables significantly differed across three groups (*p* < 0.05) (Table 1).

**Table 1:** Clinical characteristics of the variables in transfusion predictive model and key features.

**Variable**	**No-transfusion**	**Less-transfusion**	**More-transfusion**	***p-*value**
	**(*n* = 192)**	**(*n* = 127)**	**(*n* = 191)**	
Age, yr [median, (IQR)]	54.00 (44.00–73.00)	60.50 (44.50–60.5)	50.00 (39.25–60.00)	<0.001^‡^
Cause of fracture (*n*, %)				<0.001^†^
Traffic	65 (33.85)	50 (39.37)	77 (40.31)	
Grind	18 (9.38)	8 (6.30)	12 (6.28)	
Fall	26 (13.54)	16 (12.60)	59 (30.89)	
Other	83 (43.23)	53 (41.73)	43 (22.51)	
Type of tile (*n*, %)				<0.001^†^
**A**				
A1	58 (30.21)	14 (11.02)	20 (10.47)	
A2	54 (28.13)	39 (30.71)	47 (24.61)	
**B**				
B1	4 (2.08)	7 (5.51)	15 (7.85)	
B2	35 (18.23)	27 (21.26)	26 (13.61)	
B3	5 (2.60)	8 (6.30)	14 (7.33)	
**C**				
C1	14 (7.29)	5 (3.94)	15 (7.85)	
C2	5 (2.60)	7 (5.51)	7 (3.66)	
C3	17 (8.85)	20 (15.75)	47 (24.61)	
Site of fracture (*n*, %)				0.06^†^
Pubis	80 (41.67)	37 (29.13)	76 (39.79)	
Ilium	43 (22.40)	29 (22.83)	92 (48.17)	
Ischium	19 (9.90)	12 (9.45)	29 (15.18)	
Sacrum	37 (19.27)	31 (24.41)	47 (24.61)	
Synchondroses pubis	3 (1.56)	1 (0.79)	6 (3.14)	
Acetabulum	21 (10.94)	30 (23.62)	65 (32.46)	
ASA score (*n*, %)				0.02^†^
1	66 (34.38)	34 (26.77)	57 (29.84)	
2	74 (38.54)	60 (47.24)	86 (45.03)	
3	48 (25.00)	32 (25.20)	41 (21.47)	
4	3 (1.56)	0 (0.00)	6 (3.14)	
5	1 (0.52)	1 (0.79)	1 (0.52)	
Comorbidities (*n*, %)				0.182^†^
Diabetes	15 (7.81)	15 (11.81)	15 (7.85)	
Hypertension	46 (23.96)	31 (24.41)	29 (15.18)	
Other	142 (73.96)	91 (71.65)	154 (80.63)	
Hemorrhagic shock (*n*, %)	8 (4.17)	7 (5.51)	37 (19.37)	<0.001^†^
Organs injury (*n*, %)	42 (21.88)	39 (30.71)	88 (46.07)	<0.001^†^
TIFO [median, (IQR)]	4.00 (0.004–40.000)	5.833 (0.01–210.000)	7.000 (0.125–69.000)	0.096^‡^
**Therapy**				
Irontherapy (*n*, %)	15 (7.89)	23 (18.11)	38 (19.90)	<0.001^†^
Hemostasis (*n*, %)	31 (16.16)	22 (17.32)	61 (31.94)	<0.001^†^
Intraoperative cell salvage (*n*, %)	1 (0.5)	5 (3.9)	14 (7.3)	0.003^†^
Delta Hb [mean, (range)]	4.00 (−26–34)	4.86 (−42–41)	8.02 (−65–62)	0.314^*^
Preoperative SBP [mean, (range)]	127.20 (90–193)	131.82 (90–180)	122.80 (72–180)	0.005^*^
Preoperative DBP [mean, (range)]	72.73 (42–115)	72.36 (52–101)	73.31 (40–140)	0.733^*^
**Data of Lab**				
HCT [median, (95% CI)]	0.31 (0.29–0.37)	0.31 (0.30–0.33)	0.28 (0.26–0.28)	<0.001^‡^
Leukocyte [median, (IQR)]	8.96 (6.47–11.74)	8.56 (7.30–10.44)	9.15 (6.74–13.03)	0.046^‡^
PLT [median, (IQR)]	179.00 (137.50–261.50)	158.00 (126.50–213.00)	157.00 (97.25–219.25)	0.021^‡^
Neutrophil [median, (IQR)]	6.94 (5.12–9.67)	6.80 (5.28–9.01)	7.48 (5.30–11.33)	0.024^‡^
Lymphocyte [median, (IQR)]	1.02 (0.75–1.41)	1.06 (0.74–1.53)	1.00 (0.72–1.49)	0.576^‡^
Creatinine [median, (IQR)]	61.15 (53.45–76.05)	71.35 (58.88–93.78)	70.8 (54.20–94.50)	0.003^‡^
Urea [median, (IQR)]	5.84 (4.29–8.03)	6.60 (5.16–8.69)	6.27 (4.56–8.33)	0.015^‡^
Serum calcium [median, (IQR)]	2.00 (1.19–2.12)	2.00 (1.72–2.13)	1.98 (1.72–2.14)	0.947^‡^
INR [median, (95% CI)]	1.18 (1.03–1.32)	2.24 (−0.07–4.55)	1.27 (1.17–1.38)	<0.001^‡^

*TIFO, time from the injury and the first operation; SBP, systolic blood pressure; DBP, diastolic blood pressure; HCT, hematocrit; PLT, platelet; INR, international normalized ratio; ASA score, the American Society of Anesthesiologists score. Delta Hb: surgical variables included change in Hb concentration from before to after surgery*.

* *ANOVA analysis*.

† *Pearson χ^2^*.

‡ *Kruskal-Wallis*.

### Outcomes and Estimation

**Figure 3:**
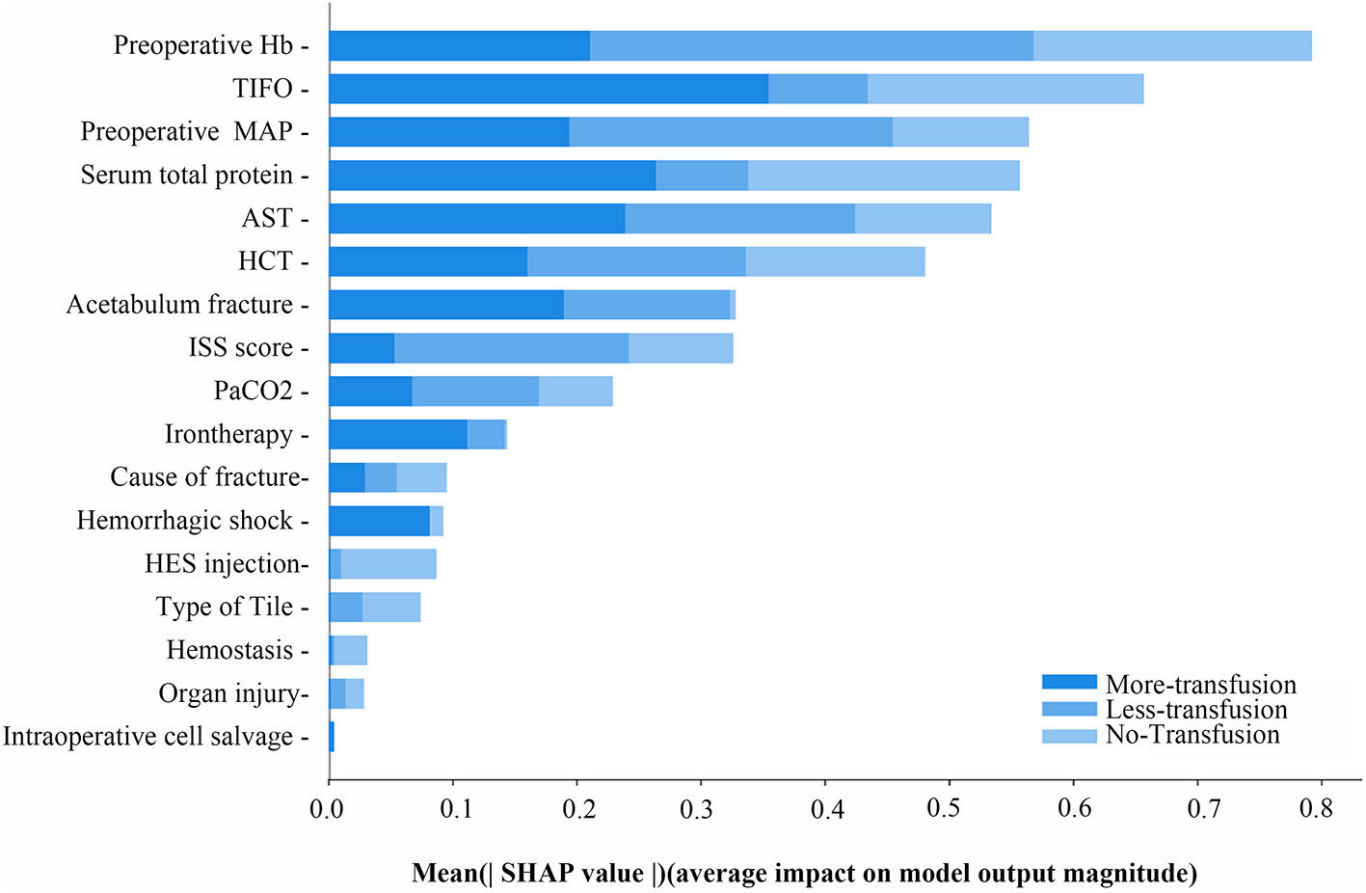
The mean SHAP value of variables in RBCs transfusion predictive model of ternary classifications. MAP, preoperative mean arterial pressure; TIFO, time from the injury and the first operation; AST, aspartate transaminase; HCT, hematocrit; ISS, injury severe score; PaCO_2_, partial pressure of carbon dioxide; HES, hydroxyethyl starch.

The XGBoost machine learning system continued to train the model until errors were minimized and accuracy was maximized, followed by the construction of an accurate RBCs predictive transfusion model. The characteristics are ordered by importance in Figure 3 with preoperative Hb, TIFO, and preoperative MAP weighted for highest importance in the final accurate transfusion predictive model.

**Figure 4:**
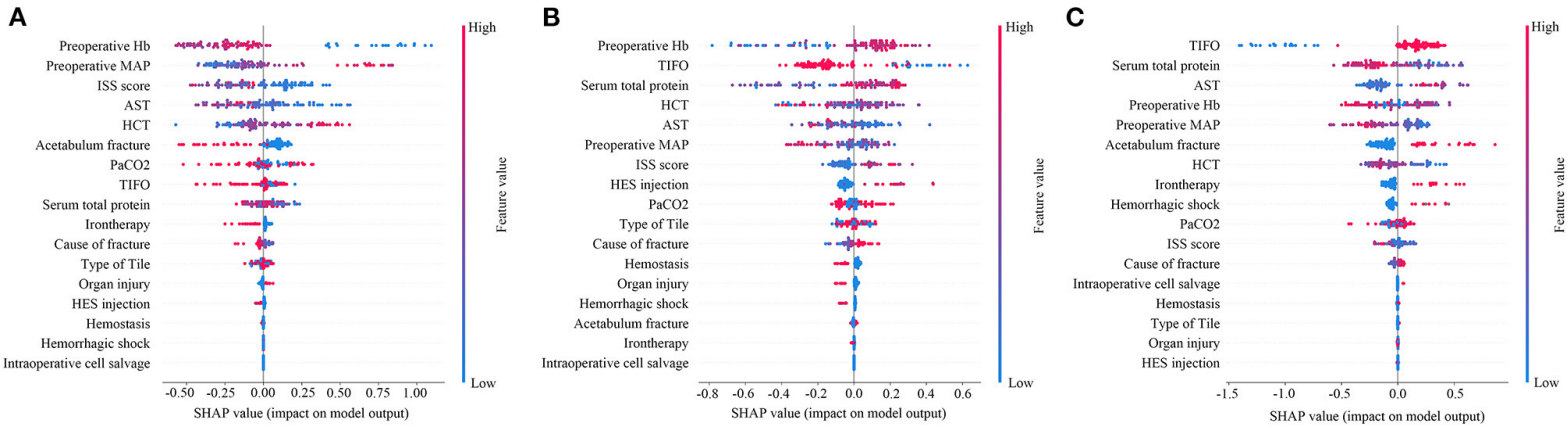
Feature importance plot for the **(A)** machine learning predictive model whether RBCs transfusion or not; **(B)** machine learning model to predict whether less RBCs transfusion (<4U) or not and **(C)** machine learning model to predict whether more RBCs transfusion (≥4U) or not. The blue and red points in each row represent participants having low to high values of the specific variable, while the x-axis gives the SHAP value, which gives the impact on the model. SHAP, Shapley Additive Explanation values; MAP, preoperative mean arterial pressure; TIFO, time from the injury and the first operation; AST, aspartate transaminase; HCT, hematocrit; ISS, injury severe score; PaCO_2_, partial pressure of carbon dioxide; HES, hydroxyethyl starch.

In order to further explore the variable weight in the machine learning model of each group, the characteristics were further analyzed by determining their SHAP values (Figure 4). SHAP values provided consistent and locally accurate attribution values for each feature within prediction mode. This is a unified approach for explaining the outcomes of any machine learning model. SHAP values evaluated the importance of the output resulting from the inclusion of feature A for all combinations of features other than A. The XGBoost algorithm based on the tree model has a unique optimization method for calculating A to increase the calculation rate. Preoperative Hb, preoperative MAP, and ISS score were the most predictive values in the machine learning model of no-transfusion, with the risk of transfusion being much lower when preoperative Hb was low (blue points), preoperative MAP was high (red points), and ISS score was low (blue points) (Figure 4A). Interestingly, in the machine learning model of less-transfusion or more-transfusion, the most predictive features were different. They were preoperative Hb, TIFO, total serum protein in the less-transfusion predictive model and TIFO, total serum protein, AST in the more-transfusion predictive model, respectively. With a high level of preoperative Hb (red points), short TIFO (blue points), and high serum total protein (red points), the risk of less-transfusion was higher (Figure 4B). Meanwhile, the long TIFO (red points), low level of serum total protein (blue points), and high level of AST (red points) were likely to be associated with more-transfusion (Figure 4C).

**Figure 5:**
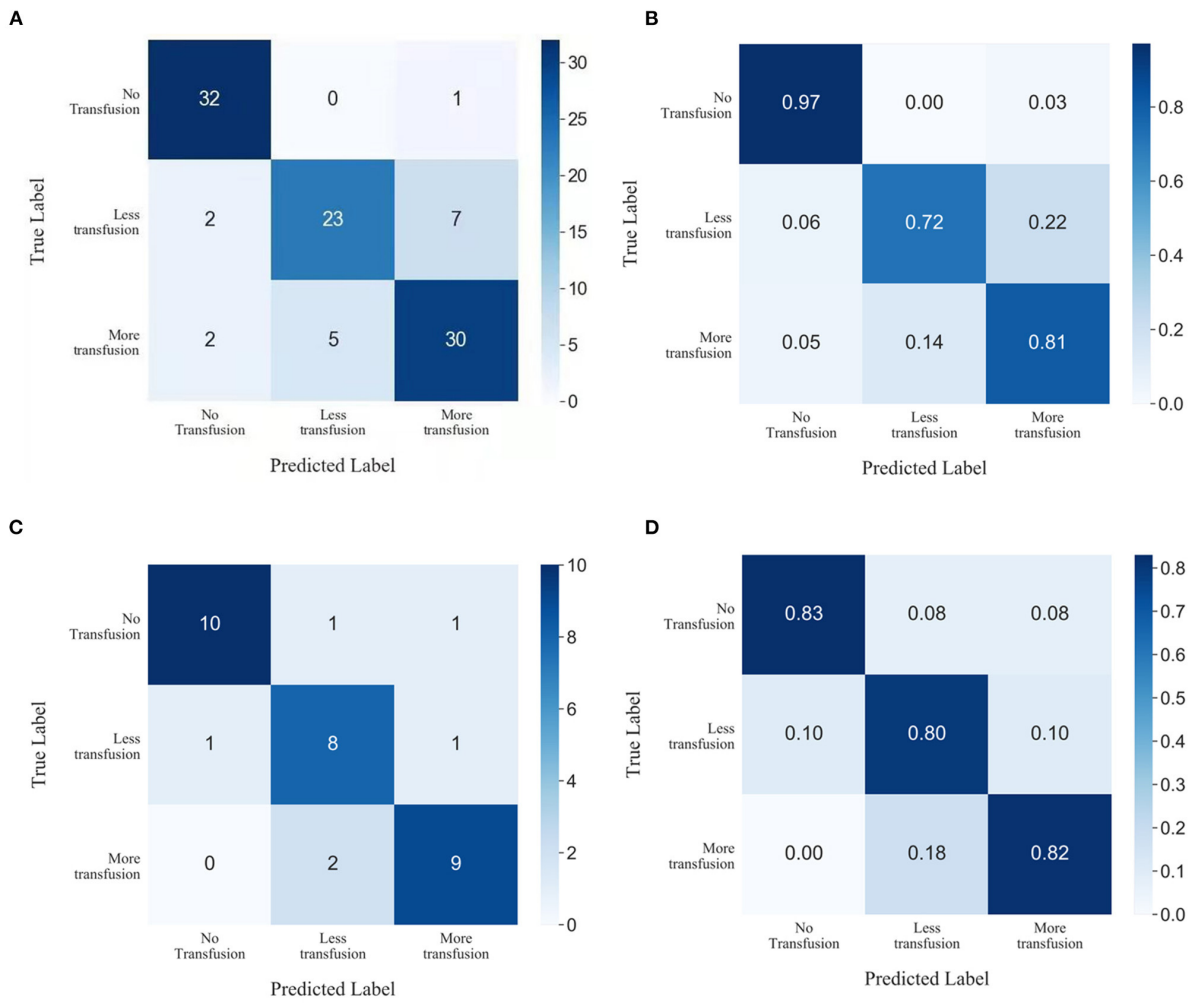
Confusion matrix showing the prediction results of XGBoost machine learning in **(A,B)** test subset and **(C,D)** prospective subset.

Performance metrics for the model based on XGBoost machine learning are presented in Table 2. The ability of this model in accurately predicting the need for perioperative RBCs transfusion (ternary classifications) in patients with pelvic fractures was compared with other transfusion predictive models and with blood preparation based on surgeons' experience. We found that the accuracy of our model was 83.34%, with a Kappa coefficient of 0.7967. This model showed the best performance relative to the ability of the surgeons to perform blood preparation based on their experience, with an accuracy of 65.94% and a Kappa coefficient of 0.5704; the random forest method had an accuracy of 82.35% and a Kappa coefficient of 0.7858; the gradient boosting decision tree method had an accuracy of 79.41% and a Kappa coefficient of 0.7742; the K-nearest neighbor method had an accuracy of 53.92% and a Kappa coefficient of 0.3341. In order to evaluate the prediction performance of XGBoost machine learning more intuitively, we have used the confusion matrix in Figures 5A,B. When using the XGBoost machine learning prediction model, the total prediction accuracy was 83.33%; the prediction accuracy was highest for No-Transfusion (96.97%) and lowest for Less-Transfusion (71.88%).

**Table 2:** The ability of different model and surgeons experience to predict the need for perioperative red blood cells transfusion in test subset (ternary classifications).

	**XGBOOST model**	**Surgeons experience**	**Random forest model**	**Gradient-boosting trees model**	**K-nearest neighbors model**
Accuracy (%)	83.34	65.94	82.35	79.41	53.92
Kappa coefficient	0.7967	0.5704	0.7858	0.7742	0.3341

### Prospective Validation

Data of 33 patients were prospectively collected for validation, of which 11 patients transfused RBCs > 4U, 10 patients received RBCs < 4U, and 12 patients did not receive any RBCs preoperatively. The total prediction accuracy of our model was 81.82% (Figures 5C,D).

### Ancillary Analyses

**Figure 6:**
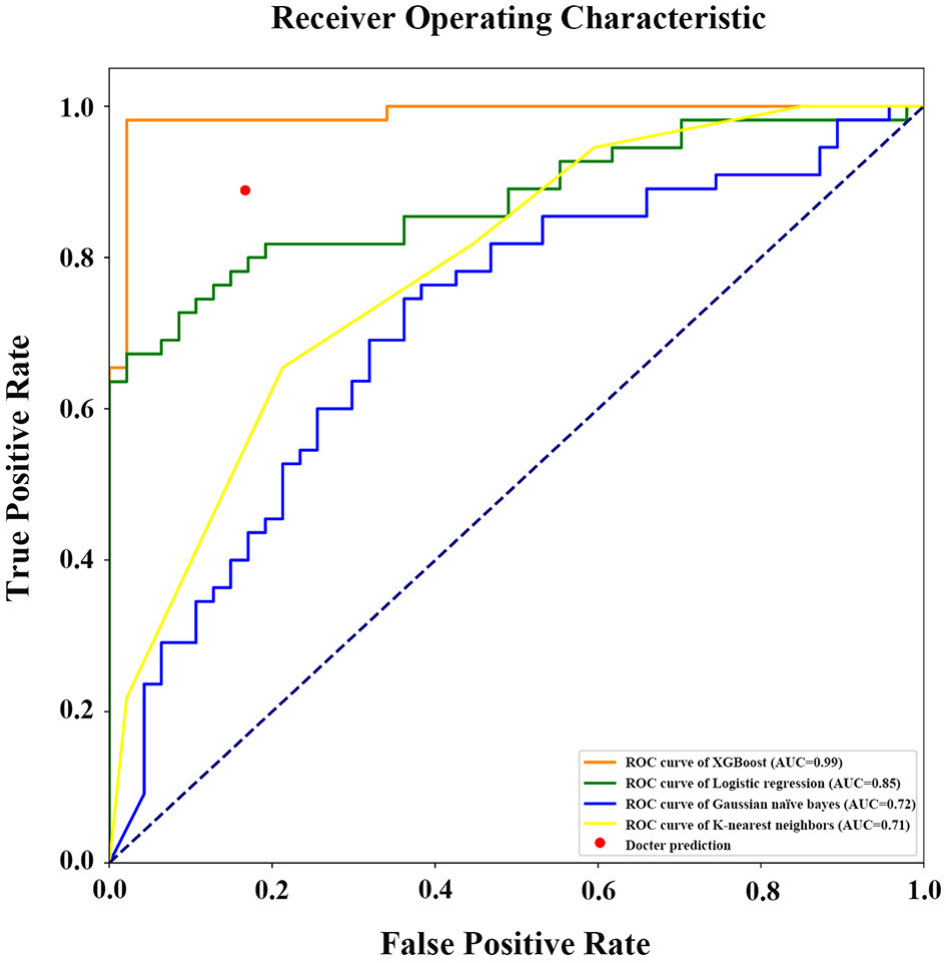
Receiver operating characteristic (ROC) curve with area under curve calculation (legend) of the different models in prediction of RBCs transfusion (binary classifications). AUC, the area under the receiver operating characteristic curve.

If the model was used to carry out a fuzzy prediction, a binary classifications model predicting whether patients did or did not require transfusions revealed that the accuracy of XGBoost was 95.13%, with an AUC of 0.99 [95% CI, 0.97–0.99], and a Youden index of 0.90. The accuracy and AUC of this model were much higher than those of other predictive models such as logistic regression, with an accuracy of 77.45%, an AUC of 0.85 (95% CI, 0.76–0.92), and Youden index of 0.53; Gaussian naïve Bayes classifier, with an accuracy of 62.75%, an AUC of 0.72 (95% CI, 0.65–0.79) and Youden index of 0.20; K-nearest neighbor, with an accuracy of 68.63%, an AUC of 0.71 (95% CI, 0.62–0.78) and Youden index of 0.35. Importantly, our model was better at predicting the need for transfusion than a model that was based on surgeons' experience that had an accuracy of 89.96% and Youden index of 0.72 (Table 3 and Figure 6).

**Table 3:** The ability of different model and surgeons experience to predict the need for perioperative red blood cells transfusion in test subset (binary classifications).

	**XGBOOST model**	**Surgeons experience**	**Logistic regression model**	**Gaussian naïve bayes classifier**	**K-nearest neighbors model**
Accuracy (%)	95.13	86.96	77.45	62.75	68.63
Youden index	0.90	0.72	0.53	0.20	0.35
AUC	0.99	/	0.85	0.72	0.71
AUC 95% CI	0.97–0.99	/	0.76–0.92	0.65–0.79	0.62–0.78
Sensitivity	0.93	0.89	0.87	0.93	0.84
Specificity	0.97	0.83	0.66	0.28	0.51

*AUC, the area under the receiver operating characteristic curve*.

## Discussion

### Generalizability

This multicenter retrospective cohort study was designed to construct a model predicting the need for perioperative RBCs transfusion in patients with pelvic fractures. The RBCs transfusion predictive model constructed by the XGBoost ensemble method achieved an accuracy of 83.34% and a Kappa coefficient of 0.7967, which represent an outstanding predictive power. To the best of our knowledge, this is the first study that used a machine learning method based on an XGBoost algorithm to accurately predict the need for RBCs transfusion (ternary classification).

### Interpretation

Although, this study attempted to make extremely accurate predictions of perioperative RBCs transfusion in patients with pelvic fractures, the outcomes of the general accuracy were not satisfactory, which may be due to the insufficient amount of data and the differences between various centers, such as the differences in surgical approaches and usage of medicines. The average dose of RBCs transfused into these patients in our study was 3.72U. It has been reported that 24% of patients with pelvic fractures require RBCs transfusions, with an average dose of 4.81U per patient (5). We chose 4U RBCs as the cut-off between less-transfusion and more-transfusion groups because a perioperative study of cardiac surgery defined massive red blood cell transfusion (MRT) as receiving at least 4U RBCs (23). The threshold of MRT was based on the increase in mortality and complications when receiving RBCs above 4U. As there is no guideline-based definition for MRT during the perioperative period, this study adopted the statement of “more-transfusion” and “less-transfusion.” Therefore, this study set the cut-off at 4 U to classify the transfusion strategy into three groups: those requiring transfusions of 0U, <4U, and ≥4U of RBCs. Although, the model in this study could not precisely predict RBCs' dose in patients with pelvic fractures, the model could accurately guide clinicians and anesthesiologists. Nonetheless, increasing the amount of data and improving its quality may result in a more precise RBCs transfusion model for patients based on the machine learning algorithm of this study.

XGBoost, the method this study used, is an ensemble method based on gradient boosted trees that have been shown to have good performance in machine learning. This method can analyze large amounts of data quickly, efficiently, and accurately, avoiding over-provisioning. Due to its outstanding advantages, it has received attention in research fields such as biomedicine (24), network security (25), and engineering (15, 16, 26–28). XGBoost has been widely accepted as the one of the models with the most impressive predictive accuracy (29). Moreover, because XGBoost used parallelism, it has been known for its ability to learn quickly and scale appropriately to the problem (30). XGBoost could provide both performance and speed, which was significant and necessary for perioperative blood transfusion. It was why we chose XGBoost instead of other algorithm. In this study, this ensemble method also showed to have a good performance in the construction of RBCs transfusion predictive model, with higher accuracy than other machine learning decision models such as random forest, gradient boosting decision tree, and K-nearest neighbor models. Sun et al. (31) predicted RBCs consumption and demand based on the XGBoost model to increase the safety of inventory management. Feng et al. (32) predicted the RBC demand in trauma patient-based XGBoost (AUC 0.71) and other decision trees. Liu et al. (33) predicted the blood transfusion after liver transplantation surgery based XGBoost (AUC 0.813). Our model showed advantages with a good balance between sensitivity and specificity in the binary prediction of perioperative transfusion risk, (whether or not transfusion is needed) in patients with pelvic fracture, as shown by its accuracy (95.1%), Youden index (0.90) and AUC (0.99). Furthermore, our research innovatively achieved ternary classification prediction and made the foundation for precise prediction of blood transfusion in the future.

The variables included in this model are easy to obtain, with preoperative Hb being the most important variable. We found that the high level of preoperative Hb was associated with a high risk of transfusion, which was not consistent with other studies. Ogbemudia et al. (34) reported that a preoperative Hb <120 g/L was associated with a 10-fold increase in transfusion requirement in patients with rheumatoid arthritis who underwent either total hip or knee arthroplasty. A retrospective study reported that preoperative lower Hb level was the independent risk factor for transfusion in total hip arthroplasty (35). These differences may be due to the longer TIFO and heavier condition (high ISS) in patients from less or more transfusion group, so they underwent a number of treatments for improving the level of Hb before the perioperative period, such as blood transfusion, iron supplementation, etc., which caused the high level of preoperative Hb in these patients. However, due to the seriousness of patients' conditions and the difficulty of operation, the blood loss during operation might be substantial, leading to the high risk of perioperative blood transfusion rate. These results suggested that even if the level of preoperative Hb was high, it was not appropriate to simply speculate the dose of transfusion during the perioperative period, but other factors needed to be considered too.

Timeliness is very important in first aid of traumatology orthopedics, where TIFO represents the time from the first trauma to surgery for patients with pelvic fractures. In our research, we suggested that the longer TIFO was strongly associated with the more transfusion, where the dose of transfused RBCs ≥4U. In many perioperative studies, perioperative RBCs transfusion was considered as an important factor causing poor prognosis (36–38). These findings suggested that emergency doctors and surgeons should reduce TIFO as soon as possible, thereby reducing perioperative allogeneic blood transfusion and improving prognosis.

Some published guidelines for patients with pelvic fracture recommend transfusing RBCs when Hb concentration ≥70 g/L (11, 39, 40). However, the modern concept of PBM points out that Hb level cannot be used solely as an RBCs transfusion strategy. This study provided a more scientific predictive model of RBCs transfusion conformed to PBM. Moreover, the remaining variables not only provided suggestions for the dose of perioperative RBCs transfusion but also proposed a way to work out a program to reduce the need for transfusion during the perioperative period for surgeons, such as iron therapy, hemostasis treatment, intraoperative cell salvage, and active first aid measures to reduce trauma-surgery time. This reflected the importance of multidisciplinary cooperation for PBM.

Most RBCs transfusion predictive models are based on traditional statistical methods, with these binary models roughly predicting the risk for transfusion (41–44). In this study, we first used the XGBoost algorithm to predict the need for perioperative RBCs transfusion (ternary classification). XGBoost-based machine learning models have many advantages over RBCs transfusion scores based on traditional statistical methods. XGBoost-based models can automatically process missing data, thus, preventing the need to make subjective assumptions about independent and dependent variables beforehand. Moreover, machine learning is more effective in dealing with complex situations compared to traditional statistical analyses (45–47). Our XGBoost-based machine learning model revealed to have the best predictive ability among all models, including the RBCs preparation according to surgeons' experience.

### Limitations

This study has several limitations. First, this was a retrospective cohort study, with inherent biases such as selection or recall bias. This model should be further used in prospective research to verify its feasibility. It is meaningful that machine learning methods in this study can continuously optimize variables, thus, providing a reliable method for many clinical predictive models. Second, this study included less data than previous studies, making it difficult to construct an extremely accurate predictive model of perioperative RBCs transfused doses in patients with pelvic fracture. Nevertheless, the present study was the first to accurately predict the risk and scope of RBCs transfusion based on the machine learning from multicenter data, which is more instructive for clinical use.

## Conclusion

This multicenter retrospective cohort study constructed an accurate model that could predict perioperative RBCs transfusion in patients with pelvic fractures. This model could simply, rapidly, and accurately predict the risk for perioperative RBCs transfusion as well as the scope of RBCs transfused doses in patients with pelvic fracture.

## Data Availability Statement

The raw data supporting the conclusions of this article will be made available by the authors, without undue reservation.

## Author Contributions

XH was responsible for writing the paper. YW, BC, YH, XW, and LC was responsible for data collecting. RG and XM was resposible for formulating plans and management. All authors contributed to the article and approved the submitted version.

## Conflict of Interest

The authors declare that the research was conducted in the absence of any commercial or financial relationships that could be construed as a potential conflict of interest.

## Publisher's Note

All claims expressed in this article are solely those of the authors and do not necessarily represent those of their affiliated organizations, or those of the publisher, the editors and the reviewers. Any product that may be evaluated in this article, or claim that may be made by its manufacturer, is not guaranteed or endorsed by the publisher.
